# Cervical EVT isolation for non-invasive fetal HLA typing in early pregnancy is limited by purity and maternal cell contamination; a methodological comparison

**DOI:** 10.3389/fimmu.2025.1575086

**Published:** 2025-05-09

**Authors:** Liseanne J. van ‘t Hof, Johanna M. Kapsenberg, Jos J.M. Drabbels, Lotte E. van der Meeren, Dave L. Roelen, Michael Eikmans, Marie-Louise P. van der Hoorn

**Affiliations:** ^1^ Department of Immunology, Leiden University Medical Center, Leiden, Netherlands; ^2^ Department of Pathology, Leiden University Medical Center, Leiden, Netherlands; ^3^ Department of Obstetrics and Gynaecology, Leiden University Medical Center, Leiden, Netherlands

**Keywords:** Trophoblast Retrieval and Isolation from the Cervix (TRIC), Fluorescence-activated cell sorting (FACS) Prenatal testing, Extravillous Trophoblast, Human Leukocyte Antigen (HLA), Preeclampsia.

## Abstract

**Introduction:**

Maternal-fetal HLA compatibility influences pregnancy outcome, including preeclampsia risk. Cervical extravillous trophoblasts (EVT) in early pregnancy provide a non-invasive source for fetal genome acquisition, potentially enabling fetal HLA typing for obstetric risk assessment. This study aimed to achieve fetal HLA typing through EVT isolation using HLA-G-coupled nanoparticle immunomagnetic separation (TRIC) and fluorescence-activated cell sorting (FACS).

**Method:**

Cervical samples from 32 pregnant women were collected by cytobrush. Saliva and umbilical cord blood (n=13) served as maternal and fetal HLA genotype controls, respectively. Cervical samples from non-pregnant women, primary cultured EVT, and cryo-sectioned term placentas served as controls for cell phenotype, protein expression, and effect of fixation. FACS and TRIC were applied to isolate EVT from maternal cells, followed by RSSO-PCR for HLA typing. EVT presence pre- and post-isolation was determined through HLA-G, β-hCG, and Cytokeratin-7 (CK-7) expression. TRIC was optimized by improving antibody-binding-efficiency, and comparing three (nano)beads types and two magnets.

**Results:**

Purity and yield of HLA-G^+^β-hCG^+^CK-7^+^ cells after TRIC failed to match pre-isolation HLA-G^+^ cell counts, despite protocol optimization. FACS revealed a fetal HLA genotype. In contrast, only the maternal HLA genotype was detected in TRIC-isolated cells.

**Conclusion:**

EVT counts and maternal cell contamination limit reliable fetal HLA typing from cervical samples. Refining non-invasive EVT isolation techniques may enable fetal HLA typing to be included in risk assessment of pregnancy complications.

## Introduction

The semi-allogeneic fetus and placenta are in contact with the maternal immune system, requiring a balance of immune activation and suppression for a successful pregnancy (1). The degree of HLA (mis)matching has been suggested to affect pregnancy outcome. High levels of HLA sharing within couples are associated with recurrent pregnancy loss (2). In contrast, a low degree of maternal-fetal HLA sharing, as seen in oocyte donation pregnancies (OD), is associated with increased risk of complications such as preeclampsia (3). Interestingly, successful OD pregnancies show a higher level of maternal-fetal HLA matching than expected by chance (4). Recently, we showed that, although there is no need for preferential selection of maternal-fetal HLA compatibility in uncomplicated autologous pregnancies, preeclamptic pregnancies show higher HLA-C, HLA class I, and total HLA matching (5). Overall, these studies suggest that maternal-fetal HLA compatibility at conception may confer a selective advantage, influencing pregnancy outcomes. Therefore, determining the HLA profile of the fetus early in gestation can contribute to improved risk management of such complications. Unfortunately, in-depth analysis of fetal DNA, and thereby HLA, during gestation is limited by current non-invasive techniques.

Current prenatal diagnostic techniques for genetic testing, including the Non-Invasive Prenatal Test (NIPT), chorionic villus sampling, and amniocentesis, all have their limitations. Among these, the latter two are invasive and carry the risk of fetal loss (6). The NIPT analyzes cell-free fetal DNA obtained from maternal plasma, distinguishing small fragments of fetal DNA from the large fraction of maternal DNA (7). The technique is clinically approved for chromosome number determination, but still has its limitations as it requires deep sequencing that is associated with high costs and low sensitivity. Moreover, the technique remains limited in its ability to support more detailed analyses, such as chromosomal mosaicism (8). Likewise, NIPT is not considered reliable for fetal HLA typing, as the technique requires sufficient genomic coverage for accurate typing (9). Moreover, the unique HLA expression profile of the circulating fetal trophoblasts – characterized by classical polymorphic HLA-C and non-classical oligomorphic HLA-E, -F, and -G, while lacking the other HLA molecules - limits RNA-based approaches, whereas whole genome sequencing remains costly (7, 10).

Extravillous trophoblasts (EVT), present in the cervical canal from early gestation, are a potential resource to obtain the full fetal genome in a non-invasive manner (11). EVT are unique in their expression of HLA-G, which is not expressed by tissues of the cervix or uterine cavity (12). This creates an exceptional opportunity to detect and isolate such cervical EVT. The technique of Trophoblast Retrieval and Isolation from the Cervix (TRIC), based on magnetic HLA-G-targeted EVT isolation, has been applied to determine fetal sex, chromosome number variants, and hemoglobin genotypes (11, 13, 14). Imudia and colleagues were the first to establish a protocol for retrieving EVT based on the simple cervical Papanicolaou (Pap) smear using a cytobrush (15). These findings along with subsequent studies, indicate that cervical samples may be applied for fetal sex determination, single nucleotide variances detection, and the isolation of intact DNA or RNA for analyzing variance and pathological protein expression profiles (13, 14, 16). The EVT yield between 5 and 20 weeks of gestation seemed unaffected by gestational age, maternal BMI, parity, and maternal age (17). Cervical EVT are suggested to resemble placental EVT, presenting opportunities to characterize molecules or pathways within placental EVT throughout gestation, including those involved in both pathophysiology and normal physiological processes (13). Yet, the biological function of the migration of EVT to the cervix remains unexplained.

Although the TRIC method appears promising and robust, the reproducibility remains uncertain as only one other research center has attempted to replicate the protocol, achieving a significantly lower success rate (18). Furthermore, established cell isolation methods, such as FACS for targeting EVT in cervical samples, have not been documented yet, nor compared to TRIC. It has been previously shown that HLA-targeted FACS can reliably separate cells present at frequencies as low as 0.01% (19).

We aimed to achieve fetal HLA typing in early pregnancy by isolating EVT from the cervix, to eventually improve risk assessment during pregnancy. This study demonstrates a critical analysis and comparison of TRIC and FACS as new potential methods for non-invasive fetal genomic analysis and its future feasibility for clinical application.

## Material and methods

### Subjects

All pregnant women (n=32) were recruited from the obstetrics department of the Leiden University Medical Center (LUMC) between May 2022 and November 2023. Cervical samples of maternal age-matched non-pregnant women (n=2) without cervical pathology were included as negative controls. Informed consent was obtained from all patients. Vital pregnancies with clinical reason for a cervical smear, non-vital pregnancies *in situ* and planned dilation or curettage were included. Exclusion criteria were twin or multiple pregnancy, molar pregnancy, previous cervical surgery, and previous cervical insufficiency. The cervical sample collection procedure in this study was the simple, standardized routine for cervical smears, performed by uniformly trained clinicians from the same department and hospital, ensuring consistency. The study protocol (nr. 22-3091) was approved by the ethics committee of the LUMC.

### Cervical and saliva sampling and processing

**Figure 1:**
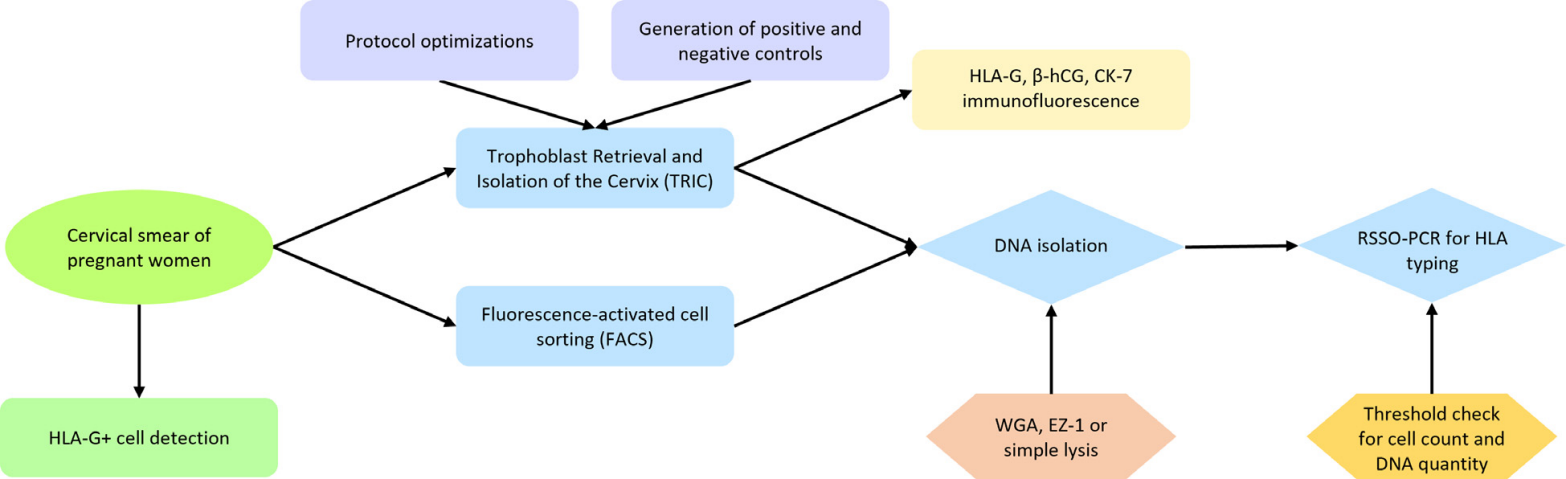
Flowchart representing the experimental workflow of this study. Cervical samples from pregnant women were collected and checked for HLA-G+ cells. These were isolated using either TRIC or FACS, followed by DNA isolation and RSSO-PCR for HLA typing. Multiple protocol optimizations and control experiments were performed to improve the result of TRIC, with results verified through immunofluorescence. Enhancements in DNA isolation methods were compared, and titration experiments were conducted to assess the requirements for reliable HLA typing.

The flowchart in **Figure 1** illustrates the experimental workflow of this study. Endocervical sampling was performed using a BD SurePath cytobrush (BD, USA), rinsed in 20 mL of ethanol-based fixative preservation fluid in a BD SurePath collection vial (BD, USA). The sample was shaken thoroughly overnight and cleared of mucus by either addition of BD density reagent buffer (BD, USA) or 600 μL of 3% acetic acid and incubated for 5 min. A small portion of the sample was taken for routine diagnostics, the remainder of the sample was transferred to the Reproductive Immunology Laboratory. The specimen was centrifuged at 400 × g at 4°C for 5 min. After washing the cells three times and resuspension in 20 mL of phosphate buffered saline (PBS), the cells were stored in 10 mL of PBS at 4°C. Maternal saliva samples were collected using the Oragene DNA OG-500 collection kit for human DNA (DNA Genotek, Canada). Umbilical cord blood (UCB) was collected in an EDTA tube after delivery. The maternal saliva and UCB samples were directly analyzed for HLA typing at the HLA laboratory of the LUMC, which is the Dutch National Reference Laboratory for Histocompatibility Testing.

Morphological analysis, immunohistochemistry and immunofluorescence are described in **Appendix I**. Details on primary and secondary antibodies used are described in **Appendix II**, **Supplementary Table 1**.

### Immunomagnetic EVT isolation

The Easysep protocol (Stemcell Technologies, Germany) was used for the immunomagnetic EVT isolation using either Phycoerythrin-(PE) or Biotin-conjugated anti-human HLA-G antibody (details in **Appendix I** and **Supplementary Table 1**). Analysis of the isolated cells was performed on a cytospin aliquot of 50 μL using the immunocytochemistry and immunofluorescence methods for HLA-G staining, as described in **Appendix I**.

The TRIC protocol was performed according to the following steps. The sample was centrifuged and resuspended in 900 μL of PBS (sterile, 4°C). Preparation of the anti–HLA-G–coated magnetic nanoparticles mixture was performed by combining 100 μL of sterile PBS, 10 μL of mouse monoclonal anti-HLA-G antibody (0.5 mg/mL Exbio, Czechia), and 10 μL of goat anti-mouse IgG magnetic nanoparticles (Clemente Associates, USA). The mix was incubated at 4°C for 15 min with regular mixing. In a comparative experiment investigating the effect of incubation duration on binding efficiency, overnight incubation at 4°C on a mixing rocker was alternatively implemented. After incubation, the mix was washed with 900 μL of PBS and placed for 10 min in an EasySep magnet, followed by resuspension in 100 μL of PBS. The mix was subsequently added to the sample. The sample-nanoparticle mixture was incubated at 4°C overnight on a mixing rocker. Unbound cells were collected after 10 min of magnetic immobilization of bound cells by either discarding or carefully aspirating the supernatant. Bound cells were collected after two rounds of washing with 1 mL of PBS and magnetizing for 10 min.

The MACS technique (Miltenyi Biotec, Germany) was performed according to the manufacturer guidelines, see details in **Appendix I**.

All anti-human HLA-G antibodies tested recognized at least the extracellular membrane-bound HLA-G1 isoform.

### Generation of positive and negative control samples

The negative-control cervical samples underwent TRIC, and immunofluorescence analysis was conducted pre- and post-isolation to examine the effect of the TRIC procedure on immunofluorescence results. Primary cultured EVT were obtained using a protocol described by Eikmans et al. (2022) (20). In short, cytotrophoblasts were isolated from placental tissue donors (n=2) who underwent termination of pregnancy between 6 and 9 weeks of gestation. Two cell lines of cytotrophoblasts were differentiated into EVT over a period of 6 days. Confirmation of EVT phenotype was achieved through specific marker expression at RNA and protein level by RT-PCR and flow cytometry, respectively. Cytospins of EVT were used for immunofluorescence analysis. EVT were added to two negative-control cervical samples at frequencies of 0.5%, 1%, and 10%. Immunofluorescence was performed before and after TRIC. Placental tissue from women with uncomplicated pregnancies who delivered at the LUMC were used as immunofluorescence controls. The placenta tissue, collected within 24h of delivery, was subsequently paraffin-embedded or frozen at -80°C. Serial sections of 4 μm were placed on glass slides for immunofluorescence analysis.

### Flow cytometry and fluorescence-activated cell sorting

Cells were washed and centrifuged in FACS buffer (1% FCS/PBS). Cells bigger than 70 μm were filtered out using a MACS Smartstrainer (Miltenyi Biotec, Germany). The sample was incubated with an antibody cocktail consisting of HLA-G-PE, ITGA1-APC, ITGA6-Pacific Blue, and CK-FITC (**Supplementary Table 1**) for 20 min at 4°C in the dark and then washed with FACS buffer. Filtration was repeated before resuspending in a final volume of 200 μL. Cells were sorted according to HLA-G expression using a BD FACSAria-III (BD, USA). Unstained cells and positive and negative CompBeads (BD, USA) were used as reference controls.

For the comparative experiment on incubation duration and binding efficiency in the TRIC protocol, HLA-G antibody binding to magnetic nanoparticles (Clemente Associates, USA) was compared between overnight and 15 min incubation. Preparation of the anti–HLA-G–coated magnetic nanoparticles was performed according to the above described TRIC protocol. Negative CompBeads were used as control. After washing in FACS buffer, the mixes were incubated with goat-anti-mouse IgG1 Alexa Fluor 647 (Invitrogen, USA) for 15 min on ice. After a final wash in FACS buffer, the mixes were analyzed on the LSR-II Flow Cytometer (BD Biosciences, USA).

### Whole genome sequencing and HLA typing

HLA-G^+^ cells from FACS were collected in 5 μL of lysis buffer (Protein kinase K/0,5% SDS). For cell lysis and protein digestion, the cells were heated at 50°C for 45 min, followed at 95°C for 10 min.

As an alternative to regular cell lysis, DNA extraction was performed through EZ-1 advanced XL (Qiagen, Germany).

For HLA typing, Reverse Sequence Specific Oligonucleotides (RSSO)-PCR (LIFECODES^®^ HLA SSO Typing Kits, Werfen, Spain) was used to type samples for HLA -A, -B, -C, -DQ, and -DP loci at the Dutch National Reference Laboratory for Histocompatibility Testing.

Detection of the Y-chromosome was performed with Real-Time PCR (RT-PCR). Reference single copy gene HCK was used alongside primers for the DYS1 locus which recognize repeated sequences on the Y-chromosome. PCR amplification was conducted on a Viia7 system (Thermo Fischer Scientific Inc., USA) with a SYBR green master mix assay (Bio-Rad, USA) over 40 cycles with 1 minute at 60°C.

To determine the minimum cell count and nanograms of DNA required for reliable detection, titration experiments with fresh, non-cultured male spleen cells (n=3) from residual material were conducted for HLA typing by RSSO-PCR and Y-chromosome detection by RT-PCR. Female spleens cells were used as negative control. The titrations included a comparison of non-fixed and fixed cells with the preservation fluid from the BD SurePath collection vial (BD, USA) as representative for cervical samples. The maximum contamination rate for reliable HLA genotype detection in cases where two genomes are mixed was analyzed for 1.25, 2.5, 5, and 10%.

Cell count was performed using a TC20 automated cell counter (Bio-Rad, USA) and confirmed by counting with a Bürker counting chamber and microscope.

## Results

### HLA-G^+^ cells were detected in all tested cervical samples, independent of the time of gestation

A total of 32 cervical samples were collected from pregnant women, including 5 samples from the first trimester (6–12 weeks), 19 samples from the second trimester (13–27 weeks), and 8 samples from the third trimester (>28 weeks). As a control for maternal and fetal HLA typing, maternal saliva was collected for 14 samples and UCB for 9 samples. Patient and sample characteristics are presented in **Supplementary Table 2**.

**Figure 2:**
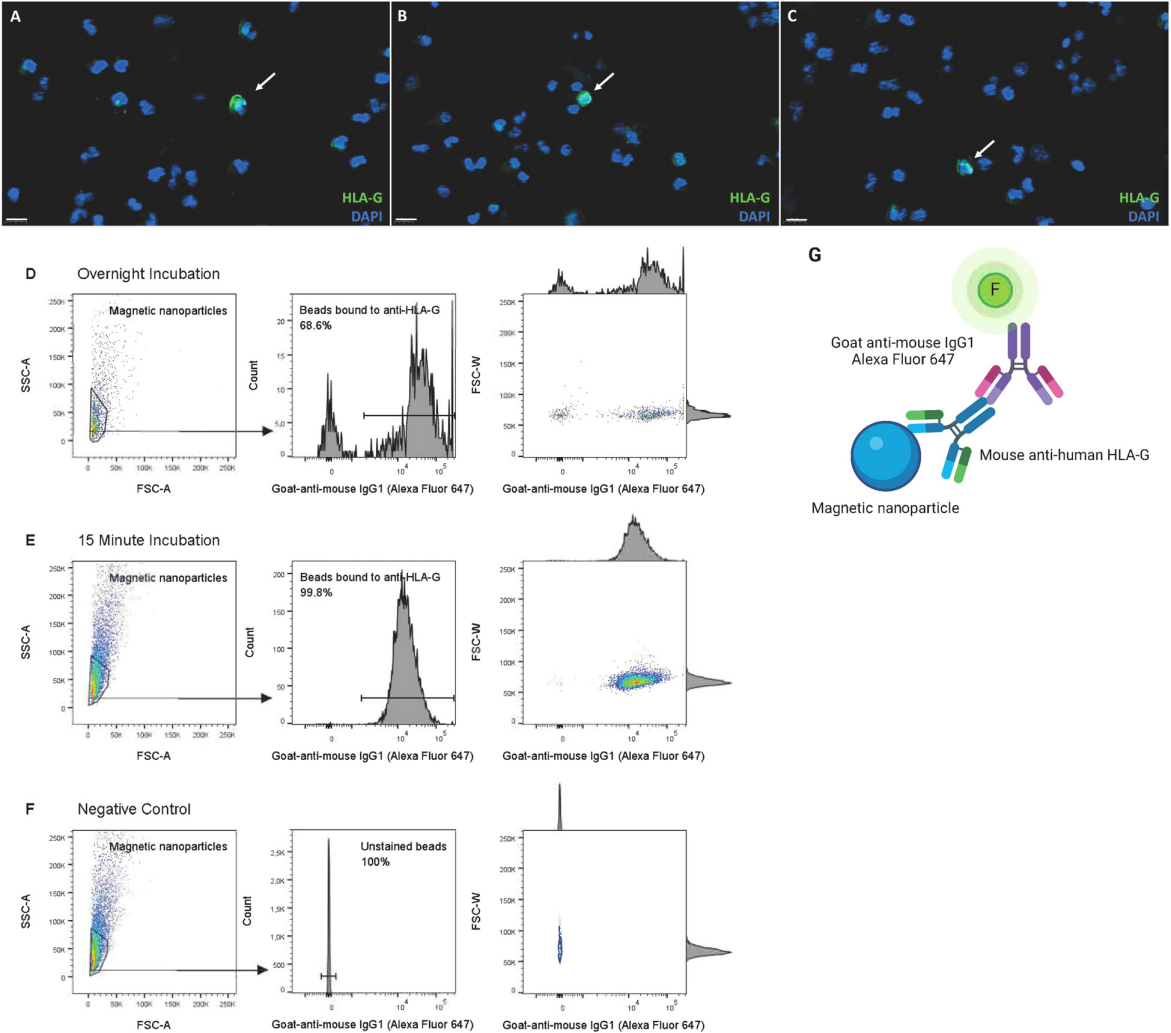
Detection of HLA-G+ EVT and optimization of anti–HLA-G–coated magnetic nanoparticles for EVT isolation. **(A–C)** Detection of EVT by HLA-G expression in cervical samples of pregnant women, through immunofluorescence, in the **(A)** first trimester (GA 6–12 weeks, n=2), **(B)** second trimester (GA 13–27 weeks, n=3) and **(C)** third trimester (GA >28 weeks, n=3), prior to isolation. Scalebar 50 μm. **(D–F)** 15 minute incubation of anti–HLA-G–coated magnetic nanoparticles is superior in binding efficiency to overnight incubation, analyzed by flow cytometry. Binding was detected with goat-anti-mouse IgG1 Alexa Fluor 647. **(G)** Schematic overview of set-up to analyze antibody-binding-efficiency by flow cytometry. This figure was created using BioRender.com.

The total number of cervical cells per sample ranged from 1x10^6^ to 34x10^6^ cells (mean of 11.19 ± 9.76x10^6^), decreasing to 0.1x10^6^ to 10x10^6^ cells (mean of 2.61 ± 2.71x10^6^) after washing and mucus removal. HLA-G^+^ cells were identified by immunofluorescence in all six tested samples (including all trimesters, **Figures 2A–C**) and by FACS in all five tested samples (2 at second trimester, 3 at third trimester). HLA-G^+^ cells were observed before isolation in every trimester of gestation, ranging from 6 to 38 weeks of gestation, with an estimated frequency of 0.01%-0.04% of total cells (**Figures 2A–C**).

### Adjustments to the TRIC protocol to increase EVT purity and yield

Isolated HLA-G^+^ EVT are expected to express β-hCG and CK-7 (13, 21). The purity and yield of β-hCG^+^CK-7^+^ cells after isolation did not correspond with the expected levels based on pre-isolation HLA-G^+^ cell counts. Therefore, attempts at optimizing the protocol were conducted, with an overview of adaptations presented in **Table 1**. Firstly, three types of antibody-magnetic (nano)bead-complexes were compared: 1) Biotin anti-human HLA-G with *RapidSpheres*, 2) PE anti-human HLA-G with *RapidSpheres*, and 3) anti-human HLA-G with goat anti-mouse IgG magnetic nanoparticles. Complex 3 yielded superior EVT counts compared to complex 1. Immunofluorescence analysis after complex 1-based magnetic isolation showed scattered HLA-G^+^ bead-bound cells among a multitude of HLA-G negative cells (**Supplementary Figure 1**). Complex 2 did not substantially enrich the HLA-G^+^ cell population compared to the pre-isolation sample.

**Table 1:** Overview of protocol adjustments and corresponding results.

Segment	Protocol adjustment	Additional details	Result	Implementation
Antibody-(nano)bead combination	Easysep protocol with PE-conjugated anti-human HLA-G antibody	N=2 PE anti-human HLA-G. (Clone 87G)	HLA-G+ cells pre-isolation: 0.01% HLA-G+ cells post-isolation: none	Omitted
	Easysep protocol with Biotin-conjugated anti-human HLA-G antibody	N=2 Biotin anti-human HLA-G. (Clone MEM G/1)	HLA-G+ cells pre-isolation: 0.01% HLA-G+ cells post-isolation: 0.3%	Omitted
	TRIC protocol with unconjugated HLA-G antibody with goat anti-mouse IgG magnetic nanoparticles	N=2 Mouse anti-human HLA-G (Clone 4H84)	HLA-G+ cells pre-isolation: 0.02% and 0.035% HLA-G+ cells post-isolation: 1.3% and 0.5% respectively	Implemented
Antibody-bead binding efficiency	Incubation duration of overnight vs. 15 minutes	Repeated once TRIC protocol with unconjugated HLA-G antibody with goat anti-mouse IgG magnetic nanoparticles	Percentage of bound antibody to beads: - overnight: 68.9% - 15 minutes: 99.7%	15 minutes implemented
Antibody-bead-sample binding	Incubation duration 1 hour vs. overnight	TRIC protocol with unconjugated HLA-G antibody with goat anti-mouse IgG magnetic nanoparticles	Not quantified, however no observed difference.	1 hour implemented
Optimization to increase cell purity	Increase washing cycles, aspirating the supernatant instead of inversion of the tube	N=3 TRIC protocol with unconjugated HLA-G antibody with goat anti-mouse IgG magnetic nanoparticles	Before; HLA-G+ cells post-isolation: 0.5-1.3% After; HLA-G+ cells post-isolation: 1.7 ‐ 2.7%	Implemented
Magnet type	Easysep magnet vs. MACS magnet (using LS columns)	N=2 Unconjugated HLA-G antibody with goat anti-mouse IgG magnetic nanoparticles	Replacement of the Easysep protocol by the MACS protocol for EVT isolation did not result in β-hCG^+^CK-7^+^ cells (n=2)	Easysep retained
Magnetic isolation	HLA-G+ cell isolation through FACS (instead of magnetic isolation)	N=5	Fetal pattern visible after FACS (2 out of 5), not after TRIC	FACS as preferred method
Cell lysis for DNA extraction	Manual DNA extraction vs. automated using EZ-1 advanced XL (Qiagen)	N=2	No difference, more samples required to determine effect	Suggestion: future research on DNA extraction optimalisation
*Fixation*	Fixed vs. non fixed samples Titration of cell number and of DNA quantity	Spleen samples, not on cervical samples Fixation with preservation fluid from the BD Surepath collection vial N=3	Reduction of signal for fixed cells compared to non-fixed cells.: - HLA typing: one titration step (1:2 dilution) - Y-chromosome: by two-fold (1 Cq increase)	Suggestion: future research with non-fixed samples if possible
*Required cell number or ng DNA*	Titration of cell number and of DNA quantity	Spleen samples, not on cervical samples N=3	Reliable detection threshold of - HLA typing: ± 1000 cells (1000–4000 depending on HLA locus) - Y-chromosome: 500 cells, 3,5ng DNA	Suggestion: future research to increase EVT count
*Contamination detection*	Titration of contamination rate	Spleen samples, not on cervical samples Mixture of two genomes N=3	Maximum contamination rate for reliable HLA genotype determination: 10%	Suggestion: future research to decrease of contamination rate

PE, phycoerythrin; FACS, fluorescence activated cell sorting; WGA, whole genome amplification.

Secondly, HLA-G antibody binding to the magnetic nanoparticles within complex 3 was further optimized by comparison of the percentage of binding after 15 min versus overnight incubation, both at 4°C; 15 min incubation was superior, yielding 99.7% compared to 68.9%. (**Figures 2D–G**).

Furthermore, literature previously reported contradictory reports on the difference in isolation yield and cell loss between MACS and Easysep magnetic isolation technologies (22, 23). Replacing the Easysep protocol with the MACS protocol for EVT isolation did not yield any β-hCG^+^CK-7^+^ cells (n=2).

Overall, post-isolation of β-hCG^+^CK-7^+^ cell purity increased, but cell yield did not, following these protocol adjustments: (1) increased wash cycles of the immobilized bound cells, (2) pipetting off the supernatant instead of inversion of the tube when using the Easysep protocol, and (3) incubation of the anti–HLA-G–coated magnetic nanoparticles mixture for 1 hour instead of overnight. In summary, by following previously published TRIC protocols we did not obtain adequate yields of EVT, despite adaptating most steps of the procedure.

### TRIC results in enrichment of HLA-G^+^β-hCG^+^CK-7^+^ cells, but does not enhance EVT purity

Successful isolation of EVT was confirmed by immunofluorescence detection of β-hCG^+^CK-7^+^ cells. Following TRIC, β-hCG^+^CK-7^+^ cells were present in 8 out of 10 samples with around 10 to 100-fold higher frequency than the pre-isolation HLA-G^+^ cells (0.3%-5.5% vs. 0.01-0.04% pre-isolation). However, despite the increased presence of EVT post-isolation, maternal cell contamination remained high (94.5-99.7%).

**Figure 3:**
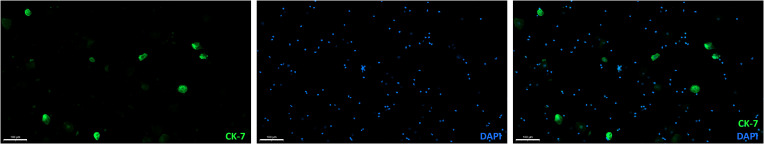
CK-7 expressing cells in a cervical sample from a non-pregnant age-matched women without cervical pathology. Overlay (right) of DAPI (middle) and CK-7 (left) immunofluorescence staining. Scalebar = 100 μm.

Importantly, CK-7^+^ cells without β-hCG expression were detected, suggesting that CK-7 is not a suitable marker for EVT detection. This was further confirmed in cervical samples of non-pregnant women, which also contained CK-7^+^ cells (**Figure 3**).

**Figure 4:**
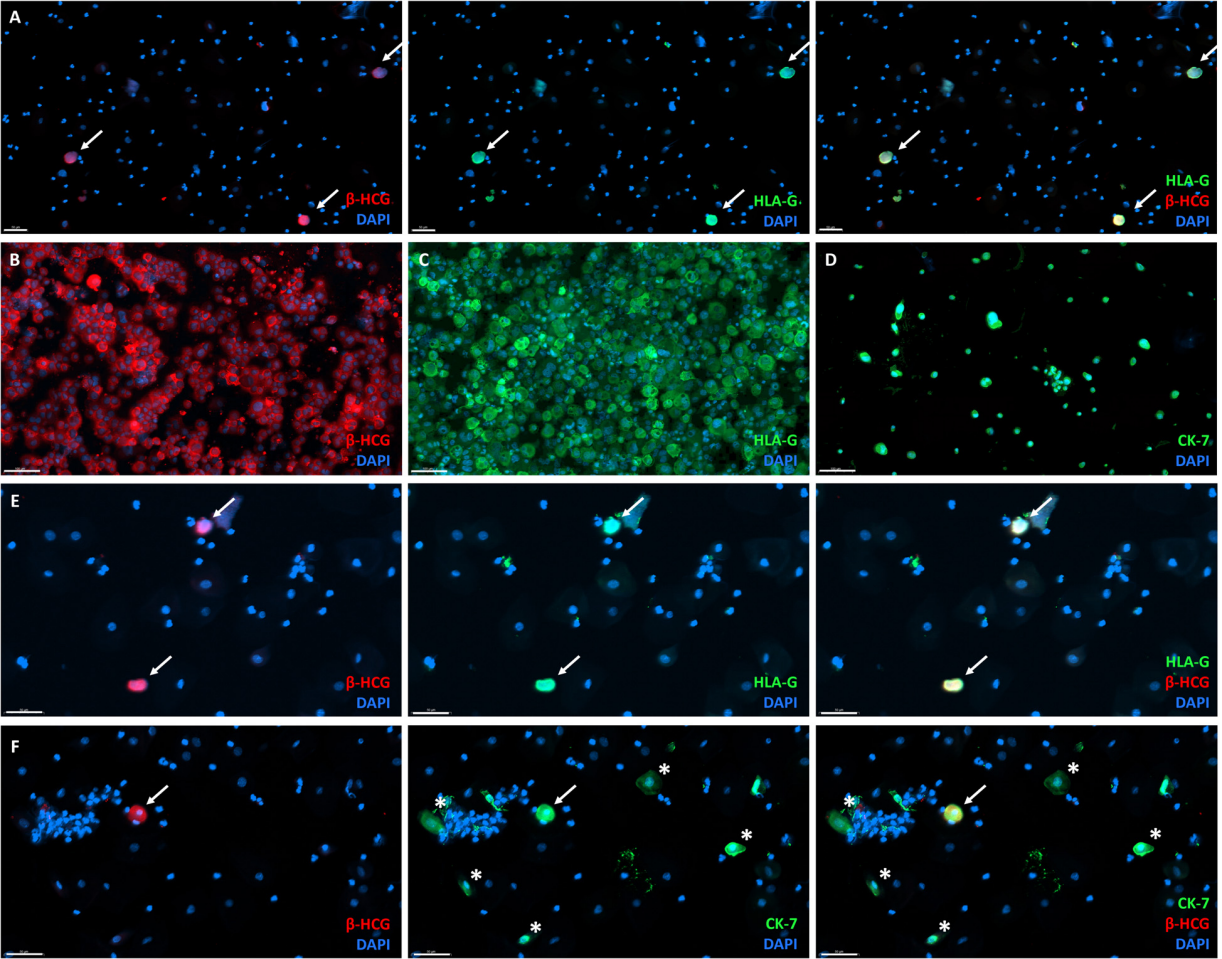
Generation of positive-control for TRIC immunohistochemistry by addition of primary cultured EVT to cervical smears from non-pregnant women. **(A)** Overlay (right) of β-hCG (left) and HLA-G (middle) immunofluorescence analysis, showing primary cultured β-hCG+HLA-G+ EVT added to non-pregnant cervical smear before isolation as an example of a generated positive-control sample. Scalebar = 50 μm. **(B–D)** Primary cultured EVT express β-hCG **(B)**, HLA-G **(C)**, and CK-7 **(D)**. Scalebar = 100 μm. **(E)** Overlay (right) of β-hCG (left) and HLA-G (middle) expression in positive control samples after TRIC. Scalebar = 50 μm. **(F)** Overlay (right) of β-hCG (left) and CK-7 (middle) expression in positive control samples after TRIC, also showing cells expressing CK-7 without β-hCG (asterix). White arrow = double positive cell (EVT). Scalebar = 50 μm.

To further determine the efficiency of the TRIC protocol, positive control samples were generated. Cultured EVT were added as 0.5%, 1%, and 10% of total cells to cervical samples of non-pregnant controls (**Figure 4A**). Expression of HLA-G, β-hCG, and CK-7 in the cultured EVT was confirmed pre-isolation (**Figures 4B–D**). Contamination of cells without HLA-G, β-hCG, and CK-7 expression remained high as the maximum amount of EVT was 0.44% (**Figures 4E, F**). Thus, the pre-isolation frequency of added EVT could not be retrieved upon enrichment and was even diminished in all samples.

Fixation of the cervical samples before TRIC did not affect the immunofluorescence results. Frozen (non-fixed) placenta sections served as controls, revealing similar expression and immunofluorescence intensity of HLA-G, β-hCG, and CK-7 as cells found in the post-isolation samples (**Supplementary Figure 2**). Additionally, the cervical samples from non-pregnant women (negative controls) showed no post-isolation expression of HLA-G or β-hCG.

### FACS revealed fetal HLA patterns, whereas TRIC yielded only maternal HLA genotype

**Figure 5:**
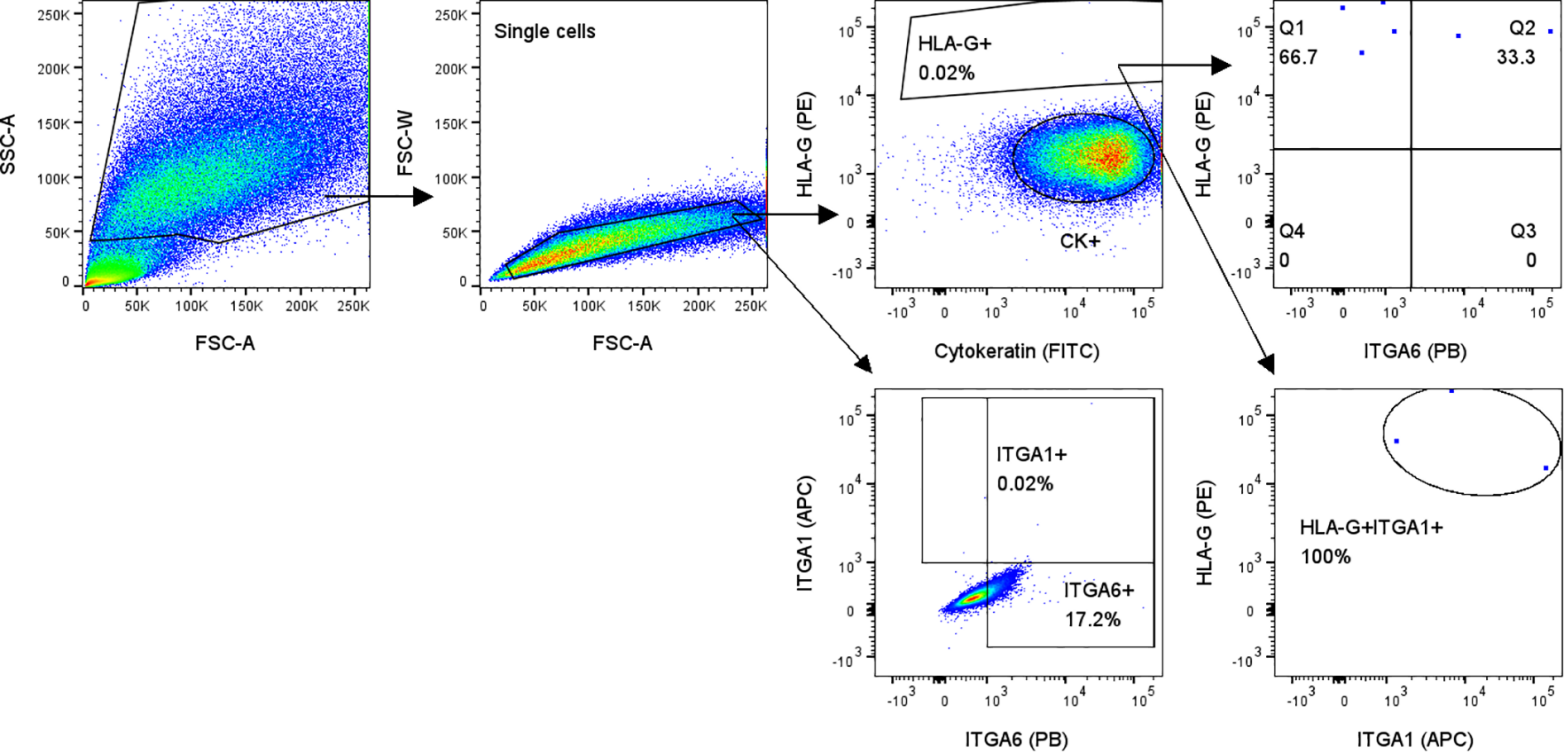
Flow cytometry analysis of cervical samples from pregnant women showed HLA-G+ cells that all expressed EVT marker ITGA1 (lower right plot). Gating on all single cells showed that ITGA6 is not specific for HLA-G+ cells (lower left plot; 17.2% of all single cells are ITGA6^+^).

Next to detection of HLA-G^high^ cells in cervical samples, FACS also confirmed large CK^+^ cells without HLA-G expression and high autofluorescence in general (**Figure 5**). HLA-G^+^ cells also expressed ITGA1, which was not present on other cells (**Figure 5**). ITGA6 was not specific for EVT, as it was expressed by 17.2% of HLA-G^-^ cells (**Figure 5**).

Importantly, in addition to the full maternal HLA genotype, a fetal HLA genotype pattern was detected in two of five FACS-sorted HLA-G^+^ samples, albeit falling just below the RSSO-PCR threshold (example in **Table 2**). Notably, distinct additional HLA alleles were detected compared to the maternal control. The additional fetal HLA proteins found within the FACS-isolated HLA-G^+^ cells were most prominent for HLA class II. In the example of **Table 2**, the DRB locus shows a third haplotype that includes a combination of the linked HLA-DRB1*04:01 and HLA-DRB4*01:01. Furthermore, HLA-DRB1*01:01 does not associate with any DRB3/4/5 allele and there is also no association between HLA-DRB1*01:01 and HLA-DRB4*01:01, all indicating a second (fetal) HLA haplotype within the sample (24).

**Table 2:** Example of fetal HLA pattern among maternal HLA genotype detection.

Sample	HLA Locus						
	A	B	C	DRB1	DRB3	DQB1	DQA1
Maternal control	A*01:01 A*02:01	B*08:01 B*51:01	C*07:01 C*14:02	DRB1*01:01 DRB1*03:01	DRB3*01:01	DQB1*02:01 DQB1*05:01	DQA1*01:01 DQA1*05:01
Cervical HLA-G+ cells	A*01:01 A*02:01	B*08:01 B*51:01	C*07:01 C*14:02	DRB1*01:01 DRB1*03:01 DRB1*04:01	DRB3*01:01 DRB4*01:01	DQB1*02:01 DQB1*05:01	DQA1*01:01 DQA1*05:01

Additional detected HLA alleles are displayed in bold, alongside to the maternal control HLA typing.

The two FACS-sorted samples without fetal HLA typing had a HLA-G^+^ cell count around 100.

In contrast, TRIC-isolated cells either yielded only a maternal HLA genotype (n=8) or an undetectable HLA genotype (n=3), despite post-isolation confirmation by immunofluorescence of enrichment of β-hCG^+^CK-7^+^ cells in 5 samples from the same isolation batch.

### HLA typing is compromised by low cell count, pre-isolation fixation, and maternal DNA contamination

For a more sensitive alternative to HLA genotyping in detecting fetal cells, we performed RT-PCR for the Y-chromosome on two cervical samples from pregnancies with male fetuses, which had resulted in 200 and 1,040 HLA-G^+^ cells after TRIC and FACS isolation, respectively. None of the samples showed positive Y-chromosome detection.

Titration experiments were conducted to compare fixed versus non-fixed cells for HLA typing and Y-chromosome analysis, aiming to determine the minimal cell count and nanograms of DNA necessary for reliable detection limits.

For detection of the Y-chromosome and HCK reference gene, reliability was achieved at a minimum cell count of 500 cells per reaction, which is equivalent to 3.5 nanograms of DNA (**Supplementary Figure 3**). A reduction of signal by two-fold (increase of 1 Cq threshold) was observed for fixed cells compared to non-fixed cells.

**Figure 6:**
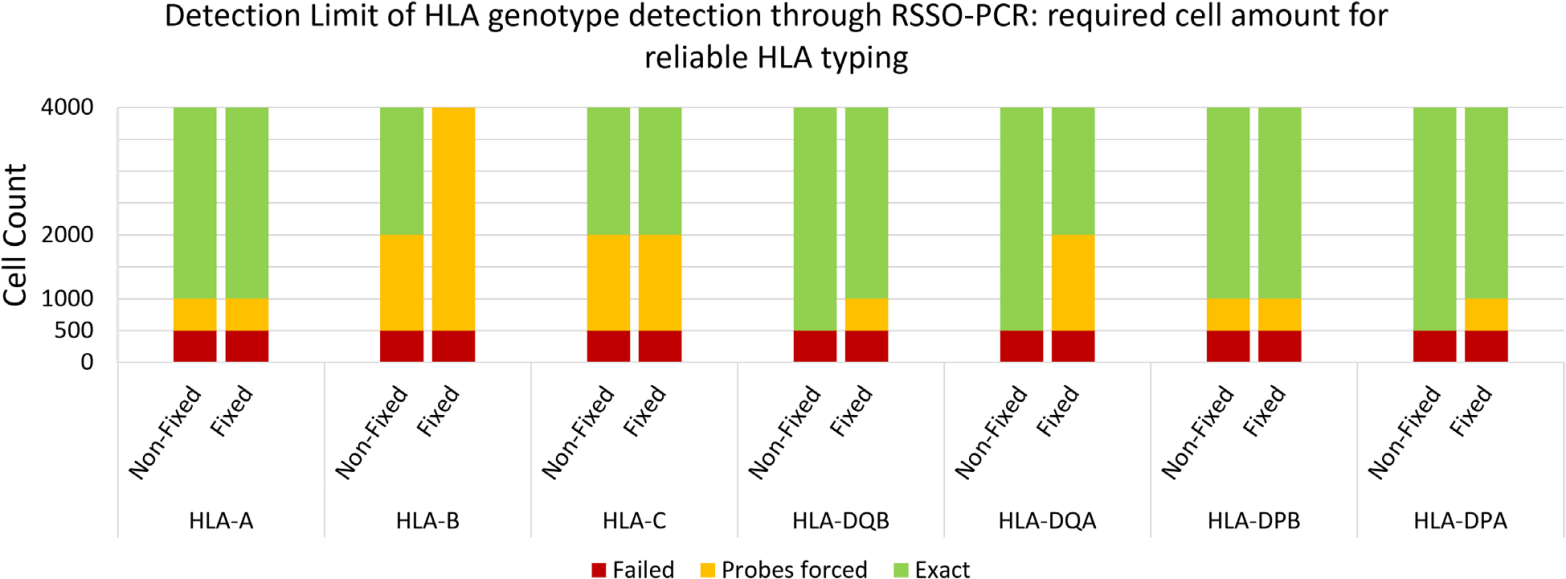
Reliable detection limit of genotype per HLA locus per cell amount. Average of 3 samples of non-fixed versus fixed male spleen cells. Probes forced = below standardized detection limit, while genotype pattern was visible and could be determined with less than 10 probes manually forced to threshold.

The minimum cell count required to reach reliable detection limits for HLA typing was 1,000 cells per locus, with sensitivity varying significantly across loci (**Figure 6**). For example, the threshold for HLA-DQB was 1,000 cells, whereas for HLA-C it remained at 4,000 cells per reaction. Fixation negatively impacted the detection threshold by lowering one titration step on average (1:2 dilution).

Considering that Y-chromosome analyses involved one primer pair and HLA class I and II analyses involved up to 5–6 primer pairs, this translated to a minimum required amount of 1,000 cells and 5,000-6,000 cells, respectively. In comparison, the maximum number of HLA-G^+^ cells isolated in this study by TRIC was 600, and by FACS was 1,051. Furthermore, we tested the reliability of target DNA detection against background DNA. Using the RSSO-PCR technique, reliable (fetal) HLA genotype determination was achievable with a maximum (maternal) contamination rate of 10% in mixed-genome samples.

## Discussion

Non-invasive fetal cell isolation for genetic testing in early gestation can contribute to enhanced obstetric risk management and improve fetal-maternal health. This study is the first to demonstrate the potential of cervical samples from pregnant women for non-invasive fetal HLA typing.

Cervical samples from pregnant women encompassed 0.01-0.04% HLA-G^+^ cells throughout all trimesters. These EVT frequencies in the cervical canal were found throughout 5–20 weeks of gestational age (17), and our results suggest this number is maintained until the third trimester. To accomplish fetal HLA typing, FACS was applied and resulted in a recognizable fetal HLA genotype. In contrast, only the maternal HLA genotype was detected in HLA-G^+^ cells isolated using TRIC. The reliable detection limit for HLA typing and Y-chromosome analysis was determined to be 1,000-4,000 cells per locus and 1,000 cells in total, respectively, with fixation negatively influencing the results. The TRIC technique failed to isolate HLA-G^+^β-hCG^+^CK-7^+^ cells without significant contamination from remaining maternal cells. Despite optimization of antibody-magnetic nanoparticle binding, increased washing cycles, and the inclusion of additional negative and positive controls, the previously published TRIC results could not be replicated. Furthermore, CK-7^+^ cells without β-hCG or HLA-G expression were detected in multiple samples, suggesting that CK-7 is not a suitable marker for EVT detection in the presence of epithelial cells. These findings suggest that EVT cell counts before isolation and the sub-optimal isolation techniques, due to maternal cell contamination, limit the effectiveness of the current RSSO-PCR HLA typing technique in achieving specific fetal HLA genotyping from cervical samples. Despite these challenges, the successful identification of a fetal HLA genotype through FACS underscores its potential as a superior alternative for isolating and analyzing EVT and fetal DNA, offering valuable prospects for obstetric risk management.

Maternal-fetal HLA compatibility has been repeatedly associated with pregnancy outcome, including recurrent pregnancy loss, preeclampsia, and birth weight (5, 25–28). Therefore, determining the fetal HLA profile early in gestation may contribute to improved risk assessment and management of such complications. Current techniques for fetal genetic analysis, such as chorionic villus sampling and amniocentesis, are invasive due to their associated risks, while the non-invasive NIPT is limited by low sensitivity and lacks the ability of in-depth DNA analysis (6–8). Furthermore, HLA typing, along with detailed analyses of different chromosomal structures (e.g. translocations, inversions, micro-duplications, micro-deletions), single nucleotide variants, and epigenetic variations, require adequate genomic coverage, which is extremely difficult given the small fetal DNA fragments present in maternal plasma (9).

This study pioneers the use of cervical samples from pregnant women for non-invasive fetal HLA typing. FACS resulted in a recognizable fetal HLA genotype. This approach demonstrates potential for clinical application as a relatively fast, non-invasive, and informative technique for fetal HLA typing. The estimated turnaround time from sample collection to test results is less than one week. This includes 2–3 days for processing, staining, and FACS-based isolation, followed by an additional day for DNA extraction and HLA typing, provided the logistics allow it.

One of the challenges yet to be surmounted is the contamination of maternal DNA in the isolated cell population. Despite HLA-G serving as a specific marker for EVT, strict selection of HLA-G^+^ cells did not provide sufficient specificity to rule out maternal DNA contamination. False-positive cell contamination stemming from factors such as autofluorescence or cell-free maternal DNA cannot be excluded as potential explanations. We could improve accuracy and gating for HLA-G^dim^ cells by including another EVT marker. For instance, we showed that all HLA-G^+^ cells also express ITGA1 (**Figure 5**), which was absent in maternal cells. Next to increasing purity, optimizing the technique is crucial to reaching the 1,000-4,000 cells per locus threshold for reliable second-field HLA typing by RSSO-PCR. With a maximum of 1,000 HLA-G^+^ cells isolated from our samples, increasing the EVT count is essential. This could be addressed by serial cervical smear collections across gestation, with pooled samples increasing the total EVT yield. This approach also allows for a comparative analysis of EVT isolation efficiency across trimesters, assessing the impact of gestational timing on HLA typing feasibility. Tandem MACS using sequential enrichment steps may further improve EVT purity and recovery. Alternatively, leveraging targeted single-cell DNA sequencing may enable reliable HLA typing despite low cell numbers. This would still require specific EVT markers, but techniques such as reverse cross-linking and nuclei isolation could help overcome challenges posed by fixation or cell clumping (29).

As a temporary alternative, focusing solely on HLA-C typing and incorporating maternal killer immunoglobulin-like receptor (KIR) typing could provide a more targeted analysis. Hiby et al. and subsequent studies by the Moffett lab found that maternal KIR AA genotypes and fetal HLA-C2 allotypes are over-represented in preeclamptic pregnancies (27, 30, 31). Indeed, we further confirmed differently distributed KIR/HLA-C genotype combinations and overall more HLA-C matches in pregnancies complicated by preeclampsia (5). While the evidence for the role of paternal HLA-C2 in preeclampsia risk is becoming increasingly substantial, clinical applications remain distant (32). Determining the fetal HLA-C2 allotype in early gestation, following further development of cervical EVT isolation, can contribute to obstetric risk management, particularly when integrated with clinical data and serological markers in mathematical prediction models. Another improvement that could enhance the accuracy and reliability of fetal HLA typing is incorporating paternal HLA as a control. This could help reliably determine fetal HLA among maternal HLA, especially for maternal-fetal HLA matching and homozygous HLA genotypes.

To isolate EVT, FACS was performed as an alternative to TRIC. While FACS had not been used before for EVT isolation from cervical samples, TRIC has been applied for fetal DNA analysis in studies by D.R. Armant’s group (13, 16, 17). In our study, TRIC failed to yield any discernible fetal HLA alleles, whereas FACS generated a fetal HLA genotype thus approaching a viable result. The absence of fetal HLA in TRIC-isolated cells raises questions about the technique’s effectiveness and reproducibility for fetal genomic analysis. Maternal contamination likely played a larger role in TRIC than FACS, with TRIC samples exceeding 90% maternal cells; far above the 10% threshold for reliable RSSO-PCR detection. Post-isolation whole genome amplification (WGA) could help to address the challenge of low EVT counts, but it may amplify maternal DNA more efficiently, potentially overshadowing fetal DNA. With enhanced fetal cell purity post-isolation, WGA could still be beneficial to elevate fetal DNA levels above the detection threshold for HLA typing.

To date, multiple attempts have been made to replicate TRIC in other research centers unaffiliated with its original development. Single-cell genotyping via laser-capture microdissection succeeded in 21 cases (33). However, the labor-intensive and low-throughput nature of laser-capture microdissection limits its applicability in routine diagnostic workflows. Van Dijk et al. (2022) replicated TRIC, but with only a 23% success rate for reliable fetal DNA testing (18). Despite a 96% success rate in EVT isolation, maternal DNA contamination also remained an insurmountable challenge. Jain et al. (2016) suggested the presence of large amounts of cell-free or exogenous maternal DNA in isolated EVT, highlighting the potential need for fetal nuclear isolation with DNase treatment (16). Remarkably, Van Dijk et al. tested various DNA isolation adaptations, including DNase treatments and Nuclear Core Complex antibodies, but these failed to yield fetal DNA (18). Additionally, they emphasized the impact of sampling depth, as previous successful TRIC studies involved women undergoing pregnancy termination, allowing more thorough sampling compared to the prospected clinical application, where miscarriage risk must be minimized to the fullest extent possible (18).

Lee et al. found that mucus removal before fixation improved EVT purity (34). Our findings also indicate that fixation negatively impacts results, doubling the required cell number for HLA typing and Y-chromosome analysis. This drawback poses challenges for clinical application, where standardized fixation is preferred over processing of fresh samples due to its flexibility and ability to preserve cell integrity. Nonetheless, considering the significance of fetal HLA typing, particularly in risk assessment for certain patient groups such as oocyte donation pregnancies, it may still be feasible to prioritize fresh sampling (4, 25). Further research is needed to optimize cervical sampling protocols, balancing reliability with clinical feasibility.

The use of EVT markers in previous TRIC studies has complicated accurate identification of these cells. While CK-7 is an effective marker in EVT cultures, its use in cervical samples potentially leads to misinterpretation as epithelial cells share lineage precursors (35). We demonstrated the presence of CK-7^+^ cells without β-hCG or HLA-G expression in cervical samples of pregnant and non-pregnant women, verifying that CK-7 is unsuitable for EVT confirmation. Likewise, morphological analysis requires caution, as we observed clearly diminished but false-positive β-hCG^+^ staining with morphological characteristics resembling epithelial cells. This phenomenon might be the cause for a discrepancy that we observed in the publication of Jain, et al. (2016), in which immunofluorescent-labelled cells after TRIC clearly resemble the morphology of epithelial cells (16). Mucus and cell clumps may cause non-specific binding, but we found that fixation does not seem to contribute to this. Valuable insights into identifying suitable unique EVT markers may stem from exploring an alternative source for non-invasive fetal DNA: peripheral maternal blood. Hatt et al. (2014) introduced CD105 and CD141 as potential markers, but noted HLA-G epitope instability in circulating cells, possibly explaining previous failures (36). Significant advancements in EVT isolation from maternal circulation may eventually lead to cervical sampling falling out favor. Nevertheless, each approach presents its challenges, such as the rarity of EVT in peripheral blood (1 EVT per 1.5x10^8^ maternal white blood cells) (36).

Future studies could leverage FACS-based cervical EVT isolation to explore its clinical utility. A case-control study of preeclamptic pregnancies could confirm increased maternal-fetal HLA (-C) compatibility as a consistent finding and the feasibility to incorporate fetal HLA typing into risk assessment protocols (5). Additionally, investigating to what extent cervical EVT resemble their decidual counterparts may provide insights into their functional relevance. This approach could pave the way for biomarker discovery or application for pregnancy complications such as recurrent pregnancy loss, chronic histiocytic intervillositis and villitis of unknown aetiology, all of which urgently require predictive tools due to the lack of effective early diagnostics (37, 38). Since EVT-immune cell interactions play a pivotal role in these disorders, studying cervical EVT could shed light on underlying mechanisms and potential therapeutic targets. Given the diagnostic value of clustered circulating trophoblasts in placenta accreta spectrum (PAS), cervical EVT isolation could provide a non-invasive screening tool for early risk assessment of PAS (39).

## Conclusion

In conclusion, our investigation into the feasibility of using cervical samples for fetal HLA typing using FACS has demonstrated its potential. A recognizable fetal HLA pattern was found after RSSO-PCR on isolated HLA-G^+^ cells, though maternal DNA contamination remains a challenge that requires further refinement. Establishing a detection threshold emphasized the importance of achieving high fetal cell purity for successful HLA typing. Meanwhile, we showed that despite efforts to optimize the technique, the replication of previous TRIC protocols remains elusive. This finding underscores the importance of replicating results, critically evaluating new techniques, and sharing these sometimes unexpected insights with the scientific community to ensure effective and reliable clinical applications. Future improvements could include refraining from using fixation, prioritizing HLA-C allotype detection, exploring additional EVT markers and utilizing single-cell techniques. Future studies could harness FACS-based cervical EVT isolation to explore its broader clinical utility, including non-invasive biomarker screening for various pregnancy complications and research on resemblance of decidual EVT, given the pivotal role of EVT-immune cell interactions.

## Data Availability

The original contributions presented in the study are included in the article/supplementary material, further inquiries can be directed to the corresponding author/s.
