# Supplementary material for: Cervical EVT isolation for non-invasive fetal HLA typing in early pregnancy is limited by purity and maternal cell contamination; a methodological comparison
Source: Front Immunol. 2025 May 9;16:1575086. doi: 10.3389/fimmu.2025.1575086 (PMC12098095; doi:10.3389/fimmu.2025.1575086)
Supplement: Supplementary file 1 [file Table1.docx]

**Appendix I – Extended Methods section**

Morphological analysis and immunohistochemistry

A 50 μL aliquot of the processed sample was centrifuged on a cytoslide (Menzel Glaser, Thermo Scientific Inc, USA) at 100 × g at 4℃ using a Shandon Cytospin 4 (Thermo Scientific Inc, USA). The amount of cells per 10 mL was estimated based on systematic cell counting using Türk’s Solution (Acetic acid gentian violet solution, Merck Life Science, Germany), a Bürker counting chamber, and a Olympus BH2 microscope (Olympus Scientific Solutions, Japan).

Morphology of the cells was examined by a May-Grünwald-Giemsa staining, in which incubation in cold methanol (1-10℃) for 3 min was followed by May-Grünwald staining-liquid for 2 min and Giemsa staining-liquid for 10 min at 20℃.

For immunohistochemistry, slides were labelled with the primary monoclonal antibody diluted in 1% bovine serum albumin(BSA)/PBS through incubation overnight at 20℃ (details in Supplemental Table 1). After washing with PBS, the secondary antibody, Anti-Mouse Envision + System HRP labelled polymer (Dako, Denmark) was incubated for 30 min at 20℃. Negative controls were isotype matched. Visualization was achieved by incubation of 50-times-diluted diaminobenzidine solution (Real DAB, Dako, Denmark) for 5 min at 20℃. Haematoxylin was used for counterstaining before slides were dehydrated and covered using mounting medium.

Immunofluorescence

The primary antibody was diluted in 1% BSA/PBS and incubated for 1 h at 20℃ (details in Supplemental Table 1). Slides were washed in PBS and the secondary antibody was incubated for 30 min at 20℃. Then 20 μL of Invitrogen Prolog Gold antifade reagent with DAPI (Thermo Fisher Scientific Inc., USA) was added. The slides were analyzed using a Pannoramic MIDI II fluorescence microscope and scanner (3DHistech, Hungary). Pre- and post-isolation immunofluorescence cell counting was automated through Histoquant software (3DHistech, Hungary). A quantification algorithm was calibrated and applied to all slides. A random selection of slides was additionally counted manually as reference control, for which the observer was blinded for the sample.

Immunomagnetic trophoblast isolation by Easysep or MACS (extended)

The Easysep protocol (Stemcell Technologies, Germany) was used for the immunomagnetic trophoblast isolation using either Phycoerythrin- (PE) or Biotin-conjugated anti-human HLA-G antibody (Supplemental Table 1). The sample was suspended in PBS-EDTA and centrifuged for 5 min at 250 × *g.* The antibody was added together with a FcR blocker, and incubated for 15 min at 20℃. Then, the samples were centrifuged for 6 min at 400 × *g*. After addition and incubation of the supplied *Selection Cocktail* for 15 min, the *RapidSpheres* solution was added and incubated for 10 min, all at 20℃. Lastly, PBS/EDTA was added and the mixture was placed in an EasySep magnet for 5 min before the supernatant was discarded. This procedure was repeated twice.

In case of the MACS protocol (Miltenyi Biotec, Germany) comparison, after incubation overnight, the sample-nanoparticle mixture was centrifuged and the supernatant was aspirated. The cell pellet was resuspended in 1 mL of MACS buffer (PBS/3%FBS/2m EDTA) and placed in a rinsed LS column within the MACS magnetic separator. The column was washed three times with 3 mL of MACS buffer and the magnetically labelled cells were eluted by taking out the separator and adding 5 mL of MACS buffer.
