# Supplementary material for: Cervical EVT isolation for non-invasive fetal HLA typing in early pregnancy is limited by purity and maternal cell contamination; a methodological comparison
Source: Front Immunol. 2025 May 9;16:1575086. doi: 10.3389/fimmu.2025.1575086 (PMC12098095; doi:10.3389/fimmu.2025.1575086)
Supplement: Supplementary file 2 [file Table2.docx]

**Appendix II – Supplemental figures and tables**


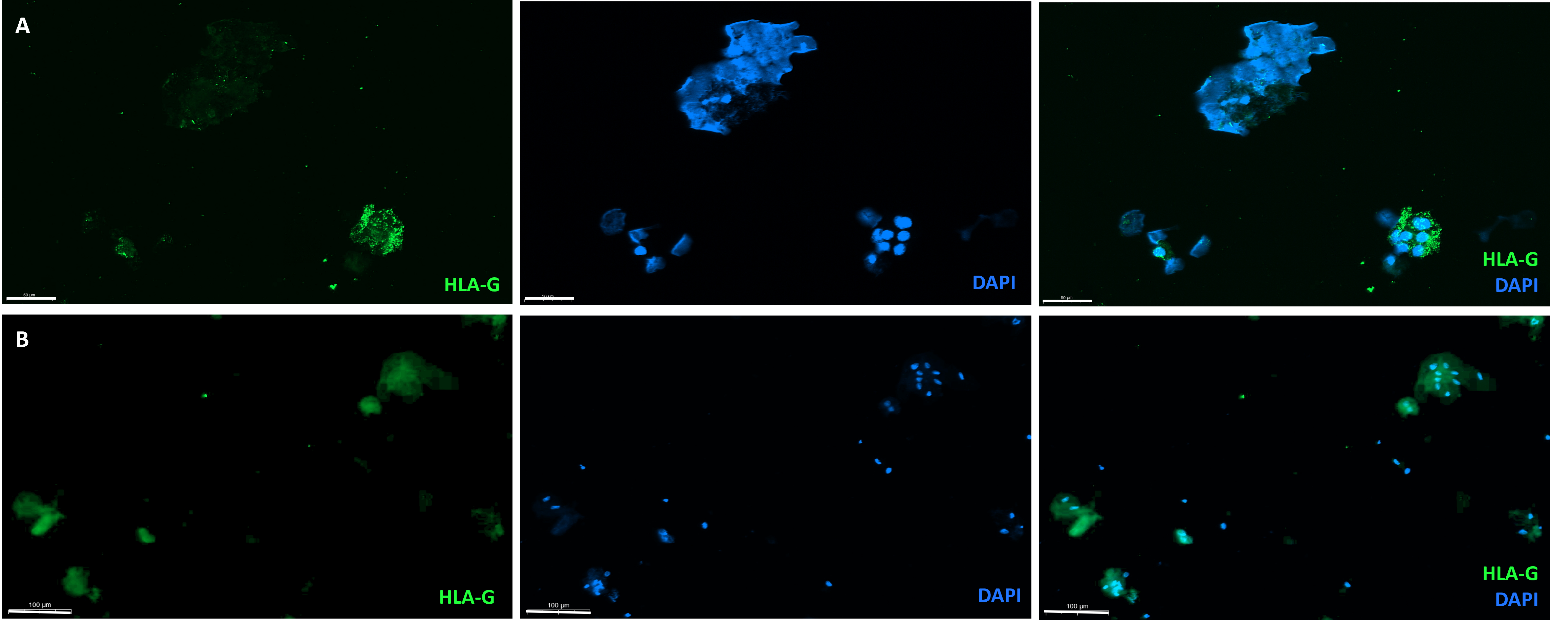


**Supplemental Figure 1**. Immunofluorescent staining of HLA-G expression in isolated cells: comparison between protocols. Overlay (right) of HLA-G+ (left) and nuclei counterstained with DAPI (middle). (A) Easysep protocol with Biotin-anti-HLA-G plus *RapidSpheres* magnetic beads. Next to HLA-G stained cells (right), a cell clump is visible with non-specific binding of DAPI to the residual mucus surrounding it. Scalebar = 50 μm. (B) TRIC protocol with anti-human HLA-G plus goat anti-mouse IgG magnetic nanoparticles. Scalebar = 100 μm. Non-bound magnetic beads appear in-between cells and adjacent to HLA-G positive and cells.


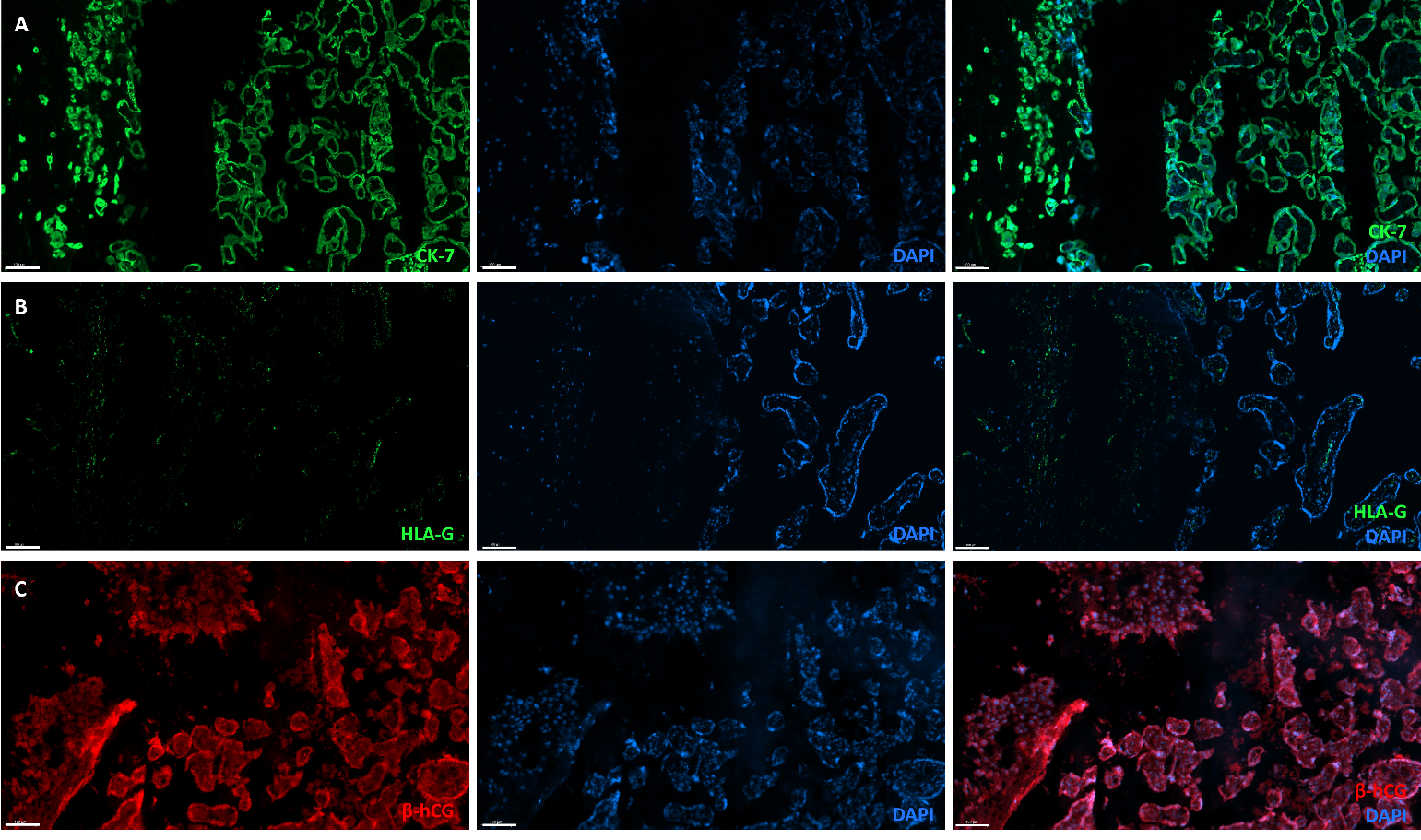


**Supplemental Figure 2**. **Immunofluorescence staining of placental tissue as non-fixed control for protein analysis.** Overlays of **(A)** CK-7, **(B)** HLA-G and **(C)** β-hCG, all counterstained with DAPI (middle). Each picture shows the decidua located on the left, and the chorionic villi on the right. Scalebar = 100 μm.

**Supplemental Figure 3.** Detection of the Y-chromosome by RT-PCR in cell-count titrated mixtures of male and female spleen cells. A titration of cell count is shown, including comparison of non-fixed and fixed cells. Fixation by preservation fluid from the BD Surepath collection vial (BD, USA). Experiment was repeated three times. HCK is the reference single copy gene used next to multicopy primers for the DYS1 locus.

**Supplemental Table 1.** Overview of antibodies used for immunohistochemistry (IHC), immunofluorescence, magnetic isolation, and fluorescence-activated cell sorting (FACS).

| **Technique** | **Target protein** | **Primary antibody** | **Dilution** | **Secondary antibody** |
| --- | --- | --- | --- | --- |
| IHC | HLA-G | Mouse anti-human HLA-G, (Clone MEM G/2, ExBio, Czechia) | 1:400 | Anti-Mouse Envision + System HRP (K4011, Dako, Denmark) |
| Immunofluorescence | HLA-G | Mouse IgG1 anti-human HLA-G, (Clone MEM G/2, ExBio, Czechia) | 1:400 | Goat anti-Mouse 488 Alexa IgG1 anti-Mouse (A21121, Invitrogen, Life technologies Europe) |
| Immunofluorescence | β-hCG | Rabbit anti-human β-hCG, polyclonal (Clone CGB7, Abcam, UK) | 1:800 | Goat anti-Rabbit 546 Alexa IgG (A11010, Invitrogen, Life technologies Europe) |
| Immunofluorescence | Cytokeratin-7 | Mouse anti-human monoclonal Cytokeratin-7 (Clone OV-TL 12/30, M7018, Dako, Denmark) | 1:100 | Goat anti-Mouse 488 Alexa IgG1 anti-Mouse (A21121, Invitrogen, Life technologies Europe) |
| Magnetic bead isolation with PE | HLA-G | PE anti-human HLA-G. (Clone 87G, 100ug/ml. Biolegend, USA) | 3 µg/mL | EasySep Human PE positive selection Kit II (Stemcell Technologies, Canada) |
| Magnetic bead isolation with Biotin | HLA-G | Biotin anti-human HLA-G. (Clone MEM G/1, Exbio, Czechia) | 3 µg/mL | EasySep Human Biotin positive selection Kit II (Stemcell Technologies, Canada) |
| Magnetic bead isolation according to TRIC protocol | HLA-G | Mouse anti-human HLA-G (Clone 4H84, 1 mg/ml, Exbio, Czechia) | 0.5 mg/mL | n.a |
| Magnetic bead isolation according to TRIC protocol | Magnetic nanoparticles | Goat anti-mouse IgG magnetic nanoparticles 250nm (Clemente Associates, USA) | 10 µL | n.a |
| FACS | HLA-G | PE mouse anti-Human HLA-G (clone 87G, Biolegend, USA) | 1:50 | n.a |
| FACS | ITGA1 (CD49a) | APC mouse anti-Human CD49a (clone TS2/7, Biolegend, USA) | 1:200 | n.a. |
| FACS | ITGA6 (CD49f) | Pacific Blue mouse anti-Human CD49f (clone GoH3, Biolegend, USA) | 1:50 | n.a. |
| FACS | Cytokeratin | FITC mouse anti-human Cytokeratin, (clone CK3-6H5, Miltenyi Biotec, Germany) | 1:200 | n.a |
| FACS | BD CompBeads | BD CompBeads Anti-Mouse Ig, κ (BD Biosciences, USA) | n.a. | n.a. |
| FACS | BD CompBeads | BD CompBeads Anti-Rat and Anti-Hamster Mouse Ig, κ (BD Biosciences, USA) | n.a. | n.a. |
| FACS | BD CompBeads | BD CompBeads Negative Control (BD Biosciences, USA) | n.a. | n.a. |

**Supplemental Table 2.** Patient and sample characteristics of all included cervical samples, including summary of results per sample.

| Sample number | Gravidity | Parity | Gestational age at sampling (weeks+days) | Trimester | Clinical reason for cervical smear | Pregnancy outcome | Maternal HLA control | Fetal HLA control | Used for/Experiment | Cell count in sample (x10^6) | Cell count after mucus clearance (x10^6) | Estimated Percentage HLA-G+ cells before isolation | Total cells after TRIC isolation (x10^6) | HLA-G+bHCG+ cells after TRIC | HLA-G+ cells through FACS | HLA typing on isolated cells | Summary of results |
| --- | --- | --- | --- | --- | --- | --- | --- | --- | --- | --- | --- | --- | --- | --- | --- | --- | --- |
| 1 | u | u | 6+2 | 1 | Vaginal bleeding | Unknown | No | No | Tests for immunofluorescence + TRIC isolation + antibody-bead complex comparison | 5 |  | 0.01% |  | 0% |  |  | Complex 3 yielded superior EVT counts compared to complex 1 and 2 |
| 2 | 2 | 0 | 14+4 | 2 | Vaginal bleeding | Elective termination, non-chromosomal | Yes | No | FACS | 23.5 |  |  |  |  | 1051 | Yes | Fetal HLA pattern detected. |
| 3 | 1 | 0 | 17+2 | 2 | Vaginal bleeding | Uncomplicated pregnancy and birth | Yes | No | TRIC Isolation + Immunofluorescence + antibody-bead complex comparison | 7 |  | 0.02% |  | 1.30% |  |  | Increase of EVT purity after isolation, however insufficient for HLA typing |
| 4 | 2 | 1 | 33+0 | 3 | unknown | Premature SC based on fetal condition | No | No | TRIC isolation + antibody-bead complex comparison |  |  |  |  |  |  |  | Complex 3 yielded superior EVT counts compared to complex 1 and 2 |
| 5 | 2 | 0 | 34+0 | 3 | Vaginal bleeding | Unknown | No | No | FACS + HLA typing | 29.5 |  | 0.04% |  |  | 72 | No | FACS panel test |
| 6 | 2 | 0 | 16+0 | 2 | Vaginal bleeding | Uncomplicated pregnancy and birth | No | No | TRIC Isolation + Immunofluorescence + antibody-bead complex comparison | 34 |  | 0.035% |  | 0.50% |  |  | Complex 3 yielded superior EVT counts compared to complex 1 and 2. Increase of EVT purity after isolation, however insufficient for HLA typing |
| 7 | 2 | 1 | 19+0 | 2 | Vaginal bleeding | Uncomplicated pregnancy and birth | No | No | TRIC Isolation + Magnet comparison + Immunofluorescence | 19 | 1.6 | 0.05% |  | 0.20% |  |  | MACS protocol did not show improvement. Increase of EVT purity after isolation, however insufficient for HLA typing |
| 8 | 1 | 0 | 29+2 | 3 | unknown | Uncomplicated pregnancy and birth | No | No | TRIC Isolation + Magnet comparison + Immunofluorescence | 6 |  |  |  | 0.30% |  |  | MACS protocol did not show improvement. Increase of EVT purity after isolation, however insufficient for HLA typing |
| 9 | 1 | 0 | 26+4 | 2 | Vaginal bleeding | Premature SC due to breech position | No | No | TRIC Isolation + Immunofluorescence | 8.6 |  |  | 0.1 |  |  |  | Increase of EVT purity after isolation, however insufficient for HLA typing |
| 10 | 5 | 1 | 7+2 | 1 | Vaginal bleeding | Spontaneous pregnancy loss | No | No | TRIC Isolation + Immunofluorescence | 1 |  | 0.01% | 0.1 | 0.70% |  |  | Increase of EVT purity after isolation, however insufficient for HLA typing |
| 11 | 1 | 0 | 30+3 | 3 | Vaginal bleeding | Uncomplicated pregnancy and birth | No | No | TRIC Isolation + Immunofluorescence |  | 0.1 |  | 0.1 | 3.30% |  |  | Increase of EVT purity after isolation, however insufficient for HLA typing |
| 12 | 1 | 0 | 19+1 | 2 | unknown | Uncomplicated pregnancy and birth | Yes | No | TRIC Isolation + HLA typing |  | 0.2 |  |  |  |  | Yes | HLA typing identical to mother |
| 13 | 4 | 3 | 38+4 | 3 | unknown | Uncomplicated pregnancy and birth | No | No | TRIC Isolation + Immunofluorescence |  |  |  | 1 | 0.60% |  |  | No EVT in isolated sample |
| 14 | 1 | 0 | 15+4 | 2 | Vaginal bleeding | Uncomplicated pregnancy and birth | No | No | TRIC Isolation + Immunofluorescence |  | 3 |  | 0.6 | 0.10% |  |  | No EVT in isolated sample |
| 15 | 1 | 0 | 8+3 | 1 | Vaginal bleeding | Gestational diabetes, uncomplicated birth | Yes | Yes | TRIC Isolation + HLA typing |  | 2.4 |  | 0.2 |  |  | Yes | HLA typing identical to mother |
| 16 | 1 | 0 | 27+0 | 2 | Vaginal bleeding | Premature birth (PPROM), cause unknown | No | No | TRIC Isolation + Immunofluorescence |  | 1.2 |  |  | 5.50% |  |  | Increase of EVT purity after isolation, however insufficient for HLA typing |
| 17 | 1 | 0 | 22+2 | 2 | Vaginal bleeding | Premature birth (PPROM), cause unknown | Yes | Yes | TRIC Isolation + HLA typing |  | 0.1 |  | 0.1 |  |  | Yes | HLA typing identical to mother (except DRB1) |
| 18 | 2 | 1 | 16+0 | 2 | Vaginal bleeding | Gestational diabetes, uncomplicated birth | Yes | Yes | TRIC Isolation + HLA typing | 3.4 | 6.3 |  | 0.5 |  |  | Yes | HLA typing identical to mother |
| 19 | 10 | 4 | 17+0 | 2 | Vaginal bleeding | Premature birth (PPROM), cause infection | Yes | No | TRIC Isolation + HLA typing |  | 4.8 |  | 0.5 |  |  | Yes | HLA typing identical to mother |
| 20 | 3 | 2 | 27+0 | 2 | Vaginal bleeding | Uncomplicated pregnancy and birth | Yes | Yes | TRIC Isolation + HLA typing | 1 |  |  | 0.1 |  |  | Yes | HLA typing identical to mother |
| 21 | 2 | 1 | 27+1 | 2 | Vaginal bleeding | Premature birth (PPROM), cause infection | No | No | TRIC Isolation + Immunofluorescence |  | 10 |  | 0.1 | 0% |  |  | Increase of EVT purity after isolation, however insufficient for HLA typing |
| 22 | 4 | 2 | 23+4 | 2 | Vaginal bleeding | Uncomplicated pregnancy, PPH | Yes | Yes | TRIC Isolation + HLA typing | 9 | 3 |  |  |  |  | Yes | HLA typing insufficient quality |
| 23 | 2 | 1 | 24+4 | 2 | Vaginal bleeding | Uncomplicated pregnancy and birth | Yes | Yes | TRIC Isolation + HLA typing | 1.5 | 0.4 |  | 0.1 |  |  | Yes | HLA typing identical to mother |
| 24 | 2 | 0 | 26+0 | 2 | Vaginal bleeding | Gestational diabetes, uncomplicated birth | Yes | Yes | TRIC Isolation + HLA typing |  | 5.3 |  | 1.5 |  |  | Yes | HLA typing identical to mother (except a few probes) |
| 25 | 5 | 0 | 23+2 | 2 | Vaginal bleeding | Gestational diabetes, uncomplicated birth | No | No | TRIC Isolation + Immunofluorescence + antibody-bead comparison |  | 5 |  |  | 1.70% |  |  | Increase of EVT purity after isolation, however insufficient for HLA typing |
| 26 | 1 | 0 | 30+2 | 3 | Vaginal bleeding | Uncomplicated pregnancy and birth | No | No | TRIC Isolation + Immunofluorescence + antibody-bead comparison |  | 1.6 |  |  | 2.70% |  |  | Increase of EVT purity after isolation, however insufficient for HLA typing |
| 27 | 4 | 1 | 17+6 | 2 | Vaginal bleeding | Uncomplicated pregnancy and birth | No | No | Tests for immunofluorescence |  | 0.6 |  |  |  |  |  | Optimalization of immunofluorescence |
| 28 | 1 | 0 | 28+4 | 3 | Vaginal bleeding | Early preeclampsia, premature birth | Yes | No | FACS + HLA typing | 6 | 1 |  |  |  | 49 | Yes | HLA typing identical to mother (early FACS stop due to techical issues) |
| 29 | 1 | 0 | 18+2 | 2 | Vaginal bleeding | Uncomplicated pregnancy, vacuum-assisted delivery, PPH | Yes | Yes | FACS + HLA typing | 15 | 0.4 |  |  |  | 75 | Yes | HLA typing identical to mother (early FACS stop due to techical issues) |
| 30 | u | u | 30+3 | 3 | unknown | Unknown | Yes (from negative cell fraction) | Yes | FACS + HLA typing | 12 |  |  |  |  | 1040 | Yes | Fetal HLA pattern detected |
| 31 | u | u | 10+1 | 1 | unknown | Unknown | Yes (from negative cell fraction) | No | TRIC Isolation + HLA typing | 5 |  |  |  |  |  | Yes | HLA typing identical to mother |
| 32 | 1 | 0 | 12+3 | 1 | Vaginal bleeding | Asymptomatic cervical cerclage, uncomplicated birth | Yes | No | TRIC Isolation + HLA typing | 15 |  |  |  | *200* |  | Yes | HLA typering class 2 identical to mother, insufficient quality for HLA class I. |
| *Negative controls* | | | | | | | | | | | | | | | | | |
| n1 | u | u | n.a. | n.a. | Follow-up low grade lesion (now Pap1a) | n.a. | n.a. | n.a. | Generation of positive control by adding cultured EVT + negative control for Immunofluorescence | 15 | 0.5/1/10% | 0.06/0.06/0.02 |  |  | n.a. | n.a. | High HLA-G-β-hCG- cell contamination (maximum EVT count after TRIC isolation 0.44%) |
| n2 | u | u | n.a. | n.a. | Vaginal itching | n.a. | n.a. | n.a. | Generation of positive control by adding cultured EVT + negative control for Immunofluorescence | 1 | 0.5/1/10% | 0.005/0.006/0.008 |  |  | n.a. | n.a. | High HLA-G-β-hCG- cell contamination (maximum EVT count after TRIC isolation 0.44%) |

SC = sectio caesarea; PPROM = preterm prelabor rupture of membranes; PPH = postpartum hemorrhage
